# Clinical Pharmacology of Oral Octreotide Capsules for the Treatment of Acromegaly

**DOI:** 10.17925/EE.2024.20.1.9

**Published:** 2024-01-22

**Authors:** Meliha Melin Uygur, Marta Villanova, Stefano Frara, Andrea Giustina

**Affiliations:** 1. Institute of Endocrine and Metabolic Sciences, Università Vita-Salute San Raffaele and IRCCS Ospedale San Raffaele, Milan, Italy; 2. Department of Endocrinology and Metabolism Disease, School of Medicine, Recep Tayyip Erdogan University, Rize, Turkey

**Keywords:** Acromegaly, efficacy, injectable somatostatin ligands, oral octreotide capsules (OOC), patient adherence, Pituitary Tumor Centers of Excellence (PTCOE), quality of life, somatostatin analogues, therapy, transient permeation enhancer

## Abstract

The primary goal of acromegaly treatment is to normalize biochemical parameters as it significantly reduces the risks of complications and comorbidities associated with the disease. First-line medical treatment is commonly represented by injectable somatostatin analogues (SRLs) after surgery. In June 2020, with the integration of Transient Permeation Enhancer® technology, oral octreotide capsules (OOCs) received regulatory approval from the US Food and Drug Administration for long-term maintenance treatment in patients with acromegaly who have responded to and tolerated treatment with octreotide or lanreotide. We reviewed the clinical pharmacological data on the development and clinical use of OOCs. The pharmacokinetic and pharmacodynamic data on OOCs showed a dose–dependent increase in octreotide levels and remarkable suppression of growth hormone secretion. The efficacy and safety of OOCs were investigated in four clinical trials conducted on patients with complete or partially controlled acromegaly. The trials resulted in the maintenance of biochemical control after switching from injectable SRLs to OOCs, with a comparable side-effect profile. Moreover, the acromegaly symptoms improved in patients on OOC. The data showed a patient preference to continue in the OOC arm for the extension phase of the trials. From the clinical pharmacological perspective, oral formulation of octreotide has the advantage of efficacy and safety with respect to injectable octreotide.

Acromegaly is a chronic disease caused by an excess of growth hormone (GH) and insulin-like growth factor 1 (IGF-1).^1,2^ Besides facial and acral changes, systemic complications lead to decreased quality of life and survival rates.^3^ The primary goal of acromegaly treatment is to normalize biochemical parameters, which significantly reduces the risks of complications and comorbidities associated with the disease.^4^ A multimodal therapeutic approach involving neurosurgery, medical therapy and radiotherapy is required to maintain these goals.^3^ The treatment of acromegaly is best determined by a multidisciplinary team of experts within the structure of a Pituitary Tumors Center of Excellence.^5–7^ The concept has recently been developed due to the need of organized care for patients with pituitary adenomas by centers of excellence where experienced neurosurgeons and pituitary-devoted endocrinologists work in collaboration with supporting units (neuroradiology, neuropathology, radiation oncology and neuroopthalmology).^5–7^

Medical therapy is mainly recommended for patients who do not achieve biochemical control after surgery, as defined by normal IGF-1 and GH levels.^1–4^ First-l ine medical treatment for acromegaly is commonly first-generation somatostatin analogues (SRLs), administered as either intramuscular (octreotide long-acting release) or deep subcutaneous injections (lanreotide depot); however, common side effects can include injection-site reactions and gastrointestinal symptoms.^3,8,9^ A significant number of patients require treatment with second-l ine and multimodal therapies, such as GH receptor antagonists (pegvisomant) or second-g eneration SRLs (pasireotide long-acting release) especially in case of resistance to first-generation SRLs.^3,10^

Improving quality of life is also one of the main goals in the management of patients with acromegaly, and it is not usually only related to biochemical control.^11–14^ Besides the symptoms of acromegaly, treatment-related side effects can also negatively impact quality of life.^9,13^ According to patient-reported outcomes surveys, chronic injections of long-acting SRLs deleteriously impact the functioning, wellbeing and daily lives of patients with acromegaly.^13,14^ Futhermore, they found that the possible reasons for persistency of active acromegaly are patients' lack of motivation on therapeutic recommendations and compliance with current medical therapy.^15^ Patients would appreciate an alternative delivery route without injections. Moreover, as medical therapy is a life-long treatment, oral administration may be an attractive alternative to improve patient acceptance and adherence.^13^

Octreotide has been used by the parenteral route due to its low and variable systemic bioavailability upon oral administration.^16–18^ In recent years, novel therapies have been studied in preclinical and clinical trials to overcome the obstacles of the medical treatment of acromegaly (*Table 1*).^19–23^ In June 2020, oral octreotide capsules (OOCs) received regulatory approval from the US Food and Drug Administration (FDA) for long-term maintenance treatment in patients with acromegaly who have responded to and tolerated treatment with octreotide or lanreotide.^4^ The aim of our paper is to review the clinical pharmacology of OOC in patients with acromegaly.

## Pharmacokinetics and pharmacodynamics of action

Oral absorption of octreotide is challenging due to enzymatic degradation and low epithelial permeability.^23^ The former could be reduced using an enteric coating, pH-modifying excipients or direct peptidase inhibitors. Moreover, peptidases do not exhibit enzyme activity in oil. An enteric capsule coating and oily suspension formulation permit an intact passage of octreotide through the stomach until the arrival of the higher pH of the small intestine. However, this hydrophilic peptide needs to dissolve in water to access the intestinal epithelium. The Transient Permeation Enhancer® (TPE®, Chiasma, Jerusalem, Israel) technology overcomes this limitation as it permits transient alteration of barrier integrity.^23,24^ It is an oily suspension composed of soluble hydrophilic microparticles of octreotide acetate, C8, and polyvinyl pyrrolidone dispersed in an oil blend, including glycerol monocaprylate and glycerol tricaprylate.^24^ Intestinal permeability experiments on rats showed that the oily suspension induced transient reorganization of tight junctions, facilitating the permeation of up to 70 kDa molecules.^25^ Dose–dependent enteral OOC absorption suppressed rat GH levels.^25^ The safety of OOC was demonstrated in monkeys after they received a daily oral administration of enteric-coated capsules for 9 months and showed no clinical or laboratory evidence of adverse findings.^25^

Oral octreotide absorption and the effect of octreotide on basal and stimulated GH secretion were evaluated in a phase I study.^26^ The study conducted on 75 healthy volunteers and 3, 10, or 20 mg oral octreotide and a single subcutaneous (sc) injection of 100 µg octreotide were administered. The study was designed to investigate oral formulations in humans and moreover, compare the pharmacokinetics of oral and injectable octreotide in heathy volunteers. The observed systemic exposure to 20 mg oral octreotide administration was similar to 0.1 mg injectable sc octreotide. The pharmacokinetic parameters after oral and parenteral octreotide dosing were comparable, and oral octreotide absorption from enteric-coated capsules was associated with a dose– dependent increase in systemic exposure. Food and proton-pump inhibitor use resulted in a reduction of 90% and 40% in bioavailability, which might be due to increased gastric pH and emptying, causing the dissolution of the pH-dependent enteric-coated capsule in the stomach. Both oral and sc octreotide treatments were well tolerated, with mild adverse events (AEs).^26^ Moreover, a single oral octreotide dose exerted a remarkable suppression of basal and GH-releasing hormone stimulated GH secretion.

In a phase III study, the pharmacokinetic profile of OOC was evaluated during the fixed-dose phase in 46 patients. Mean plasma octreotide (40 mg/d, 60 mg/d, and 80 mg/d, respectively) reached higher concentrations on 80mg/d.^19^ The mean of apparent steady state elimination half-l ife ranged from 3.19 ± 1.07 h (mean ± standard deviation) on 40 mg, to 4.47 ± 2.02 hours on 80 mg.^19^

## Clinical pharmacology

### The CH-ACM-01 trial

CH-ACM-01 (Efficacy and safety of oral octreotide for acromegaly; ClinicalTrials.gov identifier: NCT01412424), the first phase III trial (a single-arm, open-l abel study) aimed to test the efficacy and safety of OOC, was conducted in 151 patients with complete or partially controlled acromegaly (IGF-1 <1.3 the upper limit of normal (ULN) for age and an integrated GH response over 2 h of <2.5 ng/mL).^19^ The patients had received a stable dose of injectable SRL for at least 3 months. The exclusion criteria were consist of receiving GH antagonists (within 3 months of the trial) or dopamine agonists (within 2 months), or radiotherapy within the previous 10 years, or having pituitary surgery within the 6 months before screening. OOC was administered twice a day at least 4 weeks after the last SRL injection. The initial treatment dose was 40 mg/day, which escalated to 60 mg/day and then 80 mg/day until controlled IGF-1 levels were achieved. The duration of the study was approximately 13 months, including a dose-escalation period (2–5 months) and an 8–11 month fixed-dose period (core and voluntary 6-m onth extension period at 7 and 13 months, respectively). Regarding the primary endpoints (IGF-1 <1.3 x ULN for age and integrated GH<2.5 ng/mL by last observation carried forward [LOCF] imputation), 65% of enrolled patients maintained their response at the end of the core treatment period and 62% at the end of treatment (up to 13 months), compared with 88.7% at the baseline visit while patients were receiving injectable SRLs. Sensitivity analysis (using the Markov Chain Monte Carlo multiple imputation [MI]) showed a 65.6% response, consistent with primary LOCF analysis. The effect was durable, and 85% of subjects initially controlled on OOCs maintained this response for up to 13 months. Overall, 58% of patients required >40 mg OOC doses to maintain response. Baseline GH levels decreased from 0.77 ng/mL to 0.40 ng/mL within 2 hours of the first OOC dose, and remained suppressed to 0.48 ng/mL at the end of treatment. Interestingly, GH was maintained or reduced in 93% of subjects enrolled versus 96% at baseline; however, 64% achieved IGF-1 <1.3 x ULN at the end of treatment versus 91% at baseline. Moreover, 80% of subjects entering the fixed-dose phase either improved (54%) or maintained (26%) acromegaly symptoms. Of the 102 subjects completing the core treatment, 86% of patients opted to participate in the 6-months extension period -this supports a patient preference for OOC rather than injectable octreotide. The most reported AEs were gastrointestinal, neurological, and musculoskeletal. Gastrointestinal AEs mostly occurred within the first 2 months of treatment and generally resolved with treatment continuation. Hypoglycaemia and hyperglycaemia were reported respectively in 4.5% and 7% of patients, neither of which led to early discontinuation. Hepatobiliary disorders were reported in 11.6% of patients, with cholelithiasis in 7.7%. Elevation in hepatic transaminase levels and jaundice was observed in one patient, possibly related to OOC. Fifty-nine subjects discontinued treatment throught the course of the study, mainly because of treatment failure (16.8%) and AEs (14.8%).^19^

### CHIASMA OPTIMAL (Octreotide capsules versus placebo treatment in multinational centres) trial

CHIASMA OPTIMAL (Maintenance of acromegaly control in patients switching from injectable somatostatin receptor ligands to oral octreotide; ClinicalTrials.gov identifier: NCT03252353), the phase III double-blind placebo-controlled (DPC) trial aimed to evaluate the efficacy and safety of OOCs in 56 patients with acromegaly who previously demonstrated biochemical control on a stable long-acting injectable SRL.^20^ Inclusion criteria were evidence of active disease (IGF-1 ≥1.3 x ULN) prior to medical therapy and injectable SRL therapy for at least 6 months and on a stable dose for three or more months with a biochemical control (mean IGF-1 ≤1 ULN based on the average of two assessments). Exclusion criteria were off-l abel dose or dosing interval of a long-acting SRL injection; participation in previous OOC phase III clinical trials; symptomatic cholelithiasis; previous conventional or stereotactic radiotherapy of the pituitary; pituitary surgery within 6 months prior to screening; treatment with pegvisomant within 24 weeks, dopamine agonists within 12 weeks, or pasireotide within 24 weeks of screening visit. Patients were randomized to two groups of 28 patients to receive either OOC or placebo capsules for the 36-week DPC period, with an optional open-l abel extension (OLE) phase. Prior to randomization, half of the patients in both groups were on high doses of SRLs. Maintenance of biochemical control was defined as mean IGF-1 ≤1 x ULN measured at weeks 34 and 36. The primary endpoint was assessed using the nonresponse imputation (worst observation carried forward [WOCF]). In the end, the mean IGF-1 was 0.97 x ULN for the OOC group, while it increased from 0.84 to 1.69 x ULN for the placebo group. The mean integrated GH levels at week 36 were 0.6 ng/mL in the OOC group, versus 2.57 ng/mL in the placebo group. The trial resulted in the maintenance of normal IGF-1 levels in 58.2% of patients for the OOC group, versus 19.4% for the placebo group (p=0.008). Target OOC dosages at the end of the DPC period on the study drug were 40 mg in 7 patients, 60 mg in 2 patients, and 80 mg in 19 patients. The post-hoc analyses of the primary endpoint for the OOC group LOCF imputation showed that 64.3% of patients in the OOC group were biochemical responders (IGF-1 ≤1.0 x ULN) at the end of the study. Instead, among those completing the DPC period, 76.2% of patients in the OOC group were biochemical responders at the end of the study. At the end of the DPC period, GH levels were maintained (<2.5 ng/mL) in 77.7% of patients in the OOC group versus 30.4% of patients in the placebo group (p<0.001). Median time-to-l oss of IGF-1 control took 16 weeks for those on placebo; however, loss of IGF-1 control was not observed for those on OOC. The effect was durable even on stringent response cutoffs, as 92% of patients who were responders at the end of the 24 weeks titration period in the OOC group had a sustained response to the end of treatment at 9 months. Moreover, this trial suggested that patients with more severe disease requiring higher doses of injectable SRL could also respond to OOCs. Besides, patients losing biochemical control returned to prior injectable SRL treatment with a restoration of their baseline response level in about 4 weeks. Therefore, these findings suggest that patients not responding to OOCs can return to prior treatment without subsequent deterioration in biochemical control. The patient's preference for OOC therapy was confirmed because 90% of patients in the OOC group chose to remain on active treatment in the OLE phase. There were no additional safety issues identified. In fact, the observed safety profile of OOCs was consistent with the known safety profile of injectable SRL, except for the lack of injection site reactions. Overall, this study provided the basis for the approval from the FDA of OOC as the first orally delivered SRL for treatment of acromegaly.^20^

**Table 1:** Phase III studies of oral octreotide capsules for the treatment of acromegaly^19–22^

Study (N)	Study design	Inclusion criteria	Exclusion criteria	Study protocol	Biochemical response definition	Treatment duration	Primary endpoint	Imputation method for missing data for analysis of PE
CH-ACM-01,2015 (N-155)^19^	Phase III, multicentre, open-label, baseline-controlled trial	Patients with acromegaly, aged 18-75 y, treated with iSRLs on a stable dose >3 mo, with biochemical control (IGF-1 <1.3 x ULN and a serum GH level <2.5 ng/mL)	ISRL at a dosing interval >4 w; pituitary radiotherapy within 10 y; pituitary surgery within 6 mo; previous treatment with PegV within 3 mo, cabergoline within 2 mo; symptomatic cholelithiasis; any condition that may compromise study participation or significantly affect gastric acidity or emptying (e.g. bariatric surgery); clinically significant Gl, renal, or hepatic disease as determined by the investigator; current use (within 1 mo) of PPIs and current chronic use of H2RAs; pregnancy or lactation	Core treatment period: 7 mo. Dose escalation phase: at least 2 mo. Fixed dose phase: 2-5 mo. Optional extension treatment period: 6 mo. Follow-up: 2 w	IGF-1 <1.3 x LN and a serum integrated GH level <2.5 ng/mL (mean of 5 fasted GH serum concentrations, collected at 30 min intervals for 2h, 2-4h post-OOG dose)	13 mo	Maintenance of response, based on IGF-1 and GH levels	LOCF
CHIASMA OPTIMAL + OLE,2020-2022 (N-56. OOC group: 28; placebo group: 28. OLE phase: _4_0)2°A	Phase III, multicentre, randomized, DPC study	Patients with acromegaly, aged >18 y, treated with iSRLs as monotherapy for >6 mo (stable dose >3 mo), with biochemical control (IGF-1 <1.0 x LN based on the average of 2 assessments at SV1 and SV2	iSRLs at an off-label dose or dosing interval; pituitary radiotherapy, pituitary surgery within 3 mo, previous treatment with PegV within 24 w; dopamine agonists, within 12 w; pasireotide, within 24 w; patients who previously participated in OOC phase III clinical trials; patients with symptomatic cholelithiasis	DPC: 36 w: 1:1 ratio on OOC or placebo. OLE: at least 12 mo (results of 48 mo)	IGF-1 <1.0 x LN based on the average of the last two assessments: CHIASMA OPTIMAL: w 34 and 36-OLE: w 46 and 48. Definition: Responder: IGF-1 <1.0 x LN. Partial responder: IGF-1 <1.0 X LN and <1.3 X LN. Nonresponder: IGF-1 >1.3 x LN	36 w+ >12 month (optional phase, with multiple extensions of 12 mo each)	Change from baseline to end of treatment in mean IGF-1 and mean GH. Proportion of patients who maintained IGF-1 control at the end of the DPC period	WOOF Additional post-hoc analyses: LOCF
MPOWERED + COMBINATION SUB-STUDY, 2022 (N-92. OOC group: 55; ISRL group: 37. Combination sub-study: 14)^22^	Phase III, multicentre, open-label, ROT	MPOWERED: Patients with acromegaly, aged 18-75 y, treated with iSRLs for at least 6 mo (stable dose >4 mo) with biochemical control (mean IGF-1 <1.3 ULN and mean integrated GH <2.5 ng/mL at screening). Combination Sub-Study (cabergoline + OOC): failure to respond to 80 mg/d OOC for >2 w during the 26-w run-in phase, inadequate biochemical response and ineligible to enter the ROT phase on 80 mg/d OOC	iSRL at a dosing interval >8 w; pituitary radiotherapy within 5 y, pituitary surgery within 6 mo; previous treatment with PegV within 12 w; dopamine agonists, within 6 w; pasireotide within 12 w; patients who previously participated in CH-ACM-01 study; any clinically significant uncontrolled concomitant disease; symptomatic cholelithiasis	MPOWERED: Run-in phase (26 w): switched from iSRL to OOC. ROT (36 w): 3:2 ratio on OOC or iSRL. Optional open-label phase:5 y. Combination Sub-Study (cabergoline + OOC): 36 w	IGF-1 <1.3 X LN using TWA	Run-in phase + randomization: 62 w. Optional open-label phase:5 y	Proportion of participants who were maintaining their biochemical response. Sensitivity analysis—RGT: proportion of participants who were maintaining their biochemical response throughout the RGT phase in randomized participants who were biochemically controlled at start of RGT	N/A

*d = day; DPC = double-blind placebo-controlled; Gl = gastrointestinal; h = hour; H2RAs = histamine type 2 receptor antagonists; IGF-1 = insulin-like growth factor 1; ISRL = injectable somatostatin analogues; LOCF = last observation carried forward; min = minute; mo = months; N/A = not applicable; OLE = open-label extension; OOC = oral octreotide capsules; PegV = pegvisomant; PPI = proton pump inhibitor; ROT = randomized controlled trial; SV = screening visit; TWA = time-weighted average; ULN = upper limit of normal; w = weeks; WOOF = worst observation carried toward; y = years.*

### Comparison of CH-ACM-01 and CHIASMA OPTIMAL trials

Both the CH-ACM-01 and CHIASMA OPTIMAL trials investigated OOCs as maintenance therapy for patients with acromegaly who were biochemical responders receiving injectable SRLs.^19,20^ However, there are some differences between the two trials. The two most important differences between CH-ACM-01 and CHIASMA OPTIMAL were the different trial designs (open-l abel versus DPC, respectively) and definition of biochemical control (single measurement IGF-1 <1.3 x LN versus IGF ≤1.0 x ULN using an average of two visits, respectively). The imputation methods were also different, as LOCF was used in CH-ACM-01, whereas WOCF was used in CHIASMA OPTIMAL (*Table 1*). Despite these differences, OOCs demonstrated a consistent degree of biochemical response across the two trials; in fact, using LOCF imputation, 65% of patients in CH-ACM-01 maintained response during the core period and 64.3% of patients in CHIASMA OPTIMAL at the end of the DPC period. Using the WOCF imputation, maintenance of response was 53% and 58.2% in the CH-ACM-01 and CHIASMA OPTIMAL trials, respectively. Therefore, among the patients who completed the dose-adjustment period of the two trials and were stabilized on a fixed dose of OOC, most achieved a durable response and entered the voluntary extension phase.^19,20,23,27^

### Durable biochemical response and safety with oral octreotide capsules in acromegaly

The CHIASMA OPTIMAL trial continued with a 48-week OLE.^21^ Patients who completed the 36-week DPC period study both on OOC or placebo or who had predefined withdrawal criteria were eligible for OLE enrolment. The starting dose was 60 mg/day, regardless of the previous dose, with the option to increase to 80 mg/day or decrease to 40 mg/ day according to biochemical control response, safety/tolerability, and signs and symptoms of acromegaly at every 2-4 weeks of follow-up in line with pharmacokinetics of OOCs. At week 48 of the OLE, OOC dosing was 40 mg/day for 3 patients (7.5%), 60 mg/day for 10 patients (25%), and 80 mg/day for 27 patients (67.5%). The mid (60 mg) starting dose could be a useful approach to simplify the dose titration with a rapid dose adjustment scheme to achieve the target individual therapeutic dose, as the dose decreased in only 7.5% of patients at the end of the trial. The OLE of the OPTIMAL trial is ongoing. An interim analysis including results of the first 48 weeks of treatment has been recently reported, providing the first data relating to the long-term persistence of acromegaly control with OOC beyond 13 months.^21^ The biochemical response was defined similarly to the CHIASMA OPTIMAL trial (IGF-1 ≤1.0 x ULN based on the average of two assessments at weeks 46 and 48). Partial response was defined as IGF-1 >1 x ULN and <1.3 x ULN, and nonresponder was defined as IGF-1 ≥1.3 x ULN. Patients who discontinued treatment for any reason were classified as nonresponders. A total of 40 patients entered the OLE trial; half of them had been treated with OOC and half with placebo during the original DPC phase of the OPTIMAL trial. Overall, 80% of patients completed the OLE, and 90% of patients completed the study at week 48 on OOC who were the members of the previous OOC recipient group, whereas 70% of patients who had previously received placebo completed the 48-week study period. Interestingly, 18 of the 19 patients (94.7%) who were defined as responders in the DPC and enrolled in the OLE maintained biochemical control at week 48. The responder rate (using the MI approach) at week 48 of the OLE for those who received OOC during the DPC was 92.6%. Additionally, all 5 patients who received placebo during the DPC period and enrolled in the OLE as responders maintained their response at week 48. It is important to underline that these data need to be interpreted with some caution, considering that 3 of the 5 patients receiving placebo had lost biochemical control at some point during the DPC period.^20^

IGF-1 levels of patients who completed the DPC period on OOC (n=19) were stable, compared with OLE baseline and week 48 results (mean IGF-1 0.91 x ULN and 0.90 x ULN, respectively). The observed mean change in GH from OLE baseline to week 48 was 0.05 ng/mL. Moreover, this response was confirmed by comparing the mean change in IGF-1 from the DPC period baseline to OLE week 48 (IGF-1 0.81 x ULN and 0.87 x ULN, respectively). The observed mean GH change in the same period was –0.16 ng/mL.^21^

Nine patients who completed the DPC period on placebo improved their biochemical values at the end of week 48 of the OLE trial (IGF-1 1.09 x ULN to 0.87 x ULN at OLE baseline and week 48, respectively). The observed mean change in GH from the baseline of the OLE to week 48 was –0.51 ng/mL, and from the DPC baseline to OLE week 48 was 0.06 ng/ mL. All patients who completed the DPC period on placebo as complete (n=5) or partial (n=1) responders maintained their response categories at week 48 of the OLE. Notably, 2 of the 3 prior placebo recipients who were nonresponders at OLE baseline shifted to complete response by week 48 while on OOC. Of the 9 prior placebo recipients who discontinued placebo during the DPC period and were responders at OLE baseline, 2 maintained complete response, 1 shifted to partial response and 1 had missing data at OLE week 48. Five of the prior placebo recipients discontinued OOC during the OLE.^21^

No additional safety issues were reported, and the most common AEs were gastrointestinal issues. Interestingly, the incidence of AEs was lower in patients who were randomized to OOC (35%) versus placebo (60%) in the DPC period. Moreover, despite the higher starting dose in OLE, the overall incidence of AEs was lower in patients entering the OLE trial as OOC-naïve patients than in patients initiating OOC in the DPC of CHIASMA OPTIMAL trial (57.9% versus 96.45). Hyperglycaemic episodes were observed in only 6 out of 40 patients during the study.^21^

### MPOWERED study

Maintenance of biochemical response with oral octreotide and injectable SRLs therapy in patients who showed previous response with both treatments was investigated in the MPOWERED (Maintenance of response to oral octreotide compared with injectable somatostatin receptor ligands in patients with acromegaly; ClinicalTrials.gov identifier: NCT02685709) trial.^22^ This study was an open-l abel, randomized controlled, multicentre, phase III trial. Inclusion criteria were patients with acromegaly aged 18–75 years who had been treated with injectable SRLs for at least 6 months prior (on a stable dose ≥4 months) with biochemical control (mean IGF-1<1.3 ULN and mean integrated GH<2.5 ng/mL at screening). Exclusion criteria included prior off-l abel injectable SRL dosing interval longer than 8 weeks, previous participation in the CH-ACM-01 trial, pituitary radiotherapy in the past 5 years, and pituitary surgery in the past 6 months. One hundred and forty-six patients switched from injectable SRL to OOC starting from 40 mg/day and titrated up to 60 mg/day or 80 mg/day as needed during the 26-week run-in phase. As in the previous trial, the first dose of oral octreotide was administered using the routine dosing interval from the last injection. At the end of the run-in phase, 94 patients (64%) were biochemical responders (average of week 24 and 26; IGF-1 <1.3 x ULN and mean integrated GH level <2.5 ng/mL at week 24). After investigator assessment, participants with adequately controlled acromegaly were assigned for the 36 week randomized treatment phase (3:2 ratio) to OOCs at optimal dose or injectable SRL at the same dose and interval they had received previously, followed by an optional open-label phase. The primary endpoint was a non-inferiority assessment of the proportion of participants maintaining biochemical response (IGF-1 <1·3 x ULN using time-weighted average [TWA]) throughout the randomized treatment phase, using a nonresponse imputation, which defined participants who discontinued in the randomized treatment phase for any reason as a treatment nonresponder. Fourteen patients with a partial responsewhile on 80 mg/day OOC therapy during the 26-week run-in phase entered a sub-study to evaluate combination therapy with OOC and cabergoline (≤3.5 mg/week) for 36 weeks. At the end of this substudy, IGF-1 improved in most of these patients (n=12, 85.7%), suggesting the possible benefit of an all-oral treatment option without the need for any injections.

Of the 116 participants who completed the run-in phase, 92 patients entered the randomized phase, and at the end, 91% of patients in the OOC group (n=55) and 100% of patients who received injectable SRLs (n=37) maintained the biochemical response. In a sensitivity analysis of the primary endpoint that evaluated TWA response throughout the randomized treatment phase, 46 (96%) of the 48 participants receiving OOC and 36 (100%) of the 36 participants in the injectable SRL group, maintained response. Moreover, a greater proportion of patients in the OOC group had received high injectable SRL doses before baseline, and a greater proportion had tumour remnants than in the injectable SRL group. Nevertheless, the strength of the outcomes was that OOC met the primary non-inferiority endpoint, despite clinical characteristics suggestive of more active disease in the OOC group. More than 60% and 50% of patients in the OOC and injectable SRLs groups chose to continue into the optional OLE of up to 5 years, supporting the high satisfaction rate with OOC treatment.^22,28^

There were no additional safety issues, as the most common AEs in both groups were gastrointestinal. During the run-in phase, patients who responded to treatment reported decreased swelling and fatigue.^22^ Moreover, using the Acromegaly Treatment Satisfaction Questionnaire (Acro-TSQ) in the 92 patients randomized during the MPOWERED trial, 3 of 5 domains (emotional well-being, treatment convenience, and treatment satisfaction) showed significant improvement.^22,29^ The MPOWERED trial suggested that OOC could meet the non-inferiority criteria to maintain biochemical response, compared with injectable SRL treatment. TWA analysis used in this study is a clinically relevant measure of IGF-1 that represents an integrated measure of efficacy across time and can limit the noise associated with high variability. As a consequence of the trial outcomes, OOC might be a favourable alternative to injectable SRLs for many patients with acromegaly.^22^

## Conclusion

Injectable SRLs are currently considered the first-l ine medical treatment in acromegaly; however, most patients complain of side effects and lifestyle burdens.^20^ The recent development of an OOC may provide a treatment with a similar clinical pharmacological profile with respect to injectable SRLs that may present improved quality of life.^19,26^Interestingly, it was observed that acromegaly symptoms improved for the majority of the patients by switching from injectable SRLs to OOC.^19,21^ This effect could be associated with a more profound suppression of GH levels seen with OOC, compared with IGF-1 levels that was shown in the studies.^19,20^ The adverse effect profile of OOCs was similar to that of injectable forms of octreotide. Patients' preference for OOC over injectable forms during the extension studies suggests that OOC could be an promising option to address quality-of-l ife concerns related to medical treatment for acromegaly.^30^ On the other hand, it was demonstrated that injectable SRLs induced tumour shrinkage beyond antisecretory effects; whereas there is no report yet of the shrinkage effect of OOCs.^31,32^

As a result of these findings, OOC treatment could be proposed in patients who have demonstrated good biochemical response on injectable octreotide or lanreotide without a clinically significant tumor remnant, as OOC met the non-inferiority criteria compared with injectable SRL treatment.^20,21^ Based on clinical trials, the recommended initial dose of OOC is 40 mg daily, administered as 20 mg twice daily, and should be taken on an empty stomach.^22^ The dose titration up to 80 mg/day could be performed according to IGF-1 levels, and the patient's signs and symptoms assesed every 2 weeks, which allows more frequent and closer dose adjustments than injectable forms.^19,20^ In line with the data from the OLE phase of the CHIASMA OPTIMAL study, an initial dose of 60 mg might also be effective.^21^ Interestingly, some patients who responded to injectable octreotide did not maintain biochemical control when switched to OOC.^19,20^ In fact, from a clinical pharmacology perspective, optimizing the number of “persistent responders” after switching treatment still represents an important challenge. Indeed, it may pave the way to the possible use of octreotide as the first-l ine medical treatment of acromegaly on which no data are available to date.

Long-term ongoing clinical trials will help to define the role of OOC in acromegaly treatment guidelines.^21,22,28^ Also, they will provide data on the durable acceptability of twice-daily oral treatment that requires a fasting state and could be intermitted by the administration of concomitant proton pump inhibitors.
